# Prognostic Significance of Variability in Ambulatory and Home Blood Pressure from the Ohasama Study

**DOI:** 10.2188/jea.17.109

**Published:** 2007-07-18

**Authors:** Takayoshi Ohkubo

**Affiliations:** 1 Departments of Planning for Drug Development and Clinical Evaluation,; 2 Tohoku University 21st Century COE Program "Comprehensive Research and Education Center for Planning of Drug Development and Clinical Evaluation", Tohoku University Graduate School of Pharmaceutical Sciences and Medicine.

**Keywords:** Blood Pressure Determination, Blood Pressure Monitoring, Ambulatory, Cerebrovascular Accident, Population, Prospective Studies

## Abstract

Ambulatory and home blood pressure (BP) can be measured over an extended period, thus generating information about BP variability. We have monitored a Japanese general population (Ohasama) for 20 years with respect to morbidity and mortality based on ambulatory and home BP, and have demonstrated the unique prognostic significance of variability in these values. A disturbed nocturnal decline in BP is associated with cerebral infarction and heart diseases, whereas a large morning pressor surge and a large nocturnal decline in BP, which are analogous to a large diurnal increase in BP, are both associated with cerebral hemorrhage. A high BP at various times of the day is associated with different subtypes of cerebrovascular and cardiovascular disease risk. Home BP in the morning and in the evening provide equally useful information for stroke risk, whereas morning hypertension, which is that specifically observed only in the morning, might be a good predictor of stroke, particularly among individuals using anti-hypertensive medication. The BP and heart rate variabilities estimated as standard deviation measured every 30 minutes by ambulatory monitoring are independent predictors. That is, a higher short-term BP variability with a lower short-term heart rate variability leads to a worse cardiovascular prognosis. These variations in BP also bias the diagnosis and treatment of hypertension, which can be managed effectively by considering the phasic, as well as the tonic, component of BP.

The most vital information related to hypertension in clinical / epidemiologic settings is conventional (casual / clinic / office / screening) blood pressure (BP). However, several issues have recently emerged regarding the true representativeness of conventional BP, and thus other methods of measuring BP, such as ambulatory BP monitoring and self-measured BP at home (home BP) have been examined. Each method of BP measurement has unique features.^1^

We have conducted an epidemiologic survey of hypertension using ambulatory BP and home BP since 1985 in general population of Ohasama, located in the northern part of Japan (the Ohasama study). The morbidity and mortality of the Ohasama population based on ambulatory BP and home BP have been monitored for 20 years and we demonstrated the unique prognostic significance of BP variabilities derived from these measurements. This study was approved by the Institutional Review Board at Tohoku University School of Medicine and by the Department of Health of Ohasama Town.

## BLOOD PRESSURE MEASUREMENTS IN THE OHASAMA STUDY

### Ambulatory Blood Pressure

Public health nurses attached an ambulatory BP monitoring device to the participants on a weekday morning and detached the device on the following morning. Participants diarized daily activities, including the time at which they went to bed and when they arose. Ambulatory BP data were included in the analysis when the monitoring period included more than 8 waking hours (daytime) and more than 4 hours in bed (night-time). These periods were estimated from the diaries maintained by the participants. Artifactual readings during ambulatory BP monitoring were defined according to the described criteria,^2^ and were omitted from the analysis. The averages of the 24-hour, daytime, and nighttime BP values were calculated for each individual. Ambulatory BP was monitored using an ABPM-630 (Nippon Colin, Komaki, Japan), which is a fully automatic device that was preset to measure BP every 30 minutes. Although systolic BP and diastolic BP were measured by both cuff-oscillometric and microphone methods, we only used data obtained using the former method in this analysis.

### Home Blood Pressure

We used the following procedure to ascertain the accuracy of home BP. Briefly, physicians and public health nurses conducted health education classes to inform the population of the significance of home BP recording and to teach them how to measure their own blood pressure. The participants performed each step in the procedure under observation by a nurse. After verifying their ability to perform this task, participants were asked to measure their BP at home while seated once every morning within 1 hour after waking, after urination, before breakfast and after 2 or more minutes of rest, and to record the measurements for 4 weeks. If individuals were taking antihypertensive drugs, home BP was measured before medication. The participants also similarly measured their home BP once every evening just before going to bed. This scheme (multiple single measurements) was introduced to establish the most generalizable methods for home BP measurement.^3^ Home BP was measured using a semiautomatic device (HEM401C; Omron Healthcare Co., Ltd., Kyoto, Japan) based on the cuff-oscillometric principle.

### Conventional Blood Pressure

Annual health check-ups that include conventional BP measurements are available to Japanese citizens from the age of 40 years. After remaining seated at rest for at least 2 minutes, nurses or technicians measure conventional BP. In Ohasama, BP was measured twice consecutively during the health check-up, using a semi-automatic device based on the microphone method (USM700F; Ueda Electronic Work Co., Ltd., Tokyo, Japan). We used data obtained from annual check-ups that proceeded within the same time period when home BP was first initiated as part of the study protocol.

## STUDY POPULATION

Ohasama had a population of 9,400 in 1985; currently it is 6,800. Over the past 20 years, we have obtained 3,000 ambulatory BP measurements from individuals aged 20 years and over, and 5,000 home BP measurements from those aged 7 years and over, as well as outcome and information on risk factors and predictors. To prospectively investigate the association between BP levels and subsequent risk of outcomes (mortality and stroke incidence), we excluded individuals aged younger than 40 years at the time of BP measurement from the analysis because death or stroke occurrence were less common among younger people. Thus, ambulatory and home BP values were mainly prospectively analyzed in 1,542 and 1,913 participants, respectively, aged 40 or over.

## MAJOR FINDINGS OF AMBULATORY BLOOD PRESSURE

### Reference Values of Ambulatory Blood Pressure

Previous studies proposing reference values for ambulatory BP measurements derived from cross-sectional observations have been based on the statistical distribution of ambulatory BP values. We first proposed that the reference values for hypertension in 24-hour ambulatory BP measurement based on prognostic criteria should be 134/79 mmHg.^4^ These values were rounded up to 135/80 mmHg and included in the guidelines of the Japan Society of Hypertension.^5^ These values were recently modified to 130/80 mmHg according to the results of a meta-analysis of prospective studies including the Ohasama study.^6^

### Comparison of Predictive Values between Ambulatory and Conventional Blood Pressure

Ambulatory BP was more closely associated with the risk of cardiovascular mortality / stroke morbidity than conventional BP measurements.^7^^-^^9^ Masked hypertension defined as normal conventional BP and high ambulatory BP during daytime monitoring was associated with a worse prognosis for cardiovascular mortality / stroke morbidity.^10^ Systolic ambulatory BP was a better predictor of stroke than diastolic ambulatory BP and ambulatory pulse pressure.^11^

### Circadian Blood Pressure Variation

Circadian BP variation (higher and lower BP during daytime and nighttime, respectively) is a feature of both normotensive individuals and those with essential hypertension. However, circadian BP variation is diminished under several pathophysiological conditions, even in patients with essential hypertension; for example, inversion sometimes occurs, resulting in nocturnal BP elevation.^1^ Those with a normal nocturnal dip were referred to as dippers, whereas those with diminished nocturnal dipping or nocturnal BP elevation (inverted dippers) were classified as non-dippers. The term 'extreme dipper' was applied to those with a nocturnal dip of 20% or more in diurnal BP.

Non-dippers in the Ohasama study are associated with a significantly higher risk of cardiovascular mortality,^12^ independently of 24-hour ambulatory BP levels.^13^ Recent analyses of stroke incidence have further demonstrated that non-dipping is associated with cerebral infarction, whereas extreme dipping and a large morning pressor surge, which are analogous to a large diurnal increase in BP, are both associated with a risk of cerebral hemorrhage.^14^

Recent analysis using 2-h moving averages of BP (a total of 24 average BP measurements for two consecutive hours based on four BP readings taken every 30 min) to compare the predictive power of BP taken during a 24-h period given the same number of measurements have confirmed those findings; hemorrhagic stroke mortality is significantly associated with elevated daytime 2-h moving averages of systolic BP (2 h-SBP), whereas mortality due to cerebral infarction and heart disease is significantly associated with elevated night-time 2 h-SBP, indicating that high BP at various times of the day is associated with different subtypes of cerebrovascular and cardiovascular disease risk.^15^

### Blood Pressure Variability and Heart Rate Variability

Ambulatory BP monitoring provides information on variability in BP and in heart rate. We estimated the variability of BP and heart rate from the Ohasama Study as the standard deviation of the daytime or night-time average measured every 30 min. Daytime systolic ambulatory BP variability was significantly related in a linear fashion with the risk of cardiovascular mortality, whereas the risk linearly increased with the decrease in daytime and nighttime heart rate variability.16 Variabilities in both BP and heart rate are independently associated with cardiovascular mortality.^16^

### Ambulatory Arterial Stiffness Index

The ambulatory arterial stiffness index (AASI) is a novel reflection of arterial stiffness defined as 1 minus the regression slope of diastolic over systolic BP in individuals, which can be determined from 24-h ambulatory BP recordings. In the Ohasama study, AASI was significantly associated with risk for cardiovascular and stroke mortality in a U-shaped fashion, whereas pulse pressure did not yield any prognostic information.^17^

## MAJOR FINDINGS OF HOME BP

### Reference Values of Home Blood Pressure

Previous studies proposing reference values for home BP measurements derived from cross-sectional observations were based on the statistical distribution of home BP values. We first proposed that the reference values for hypertension of home BP measurement based on prognostic criteria should be 137/84 mmHg.^18^ These values were rounded to 135/85 mmHg and were subsequently included in several guidelines.^1^^, ^^2^^, ^^5^

### Comparison of Predictive Values between Home and Conventional Blood Pressure

Home BP was more closely associated with the risk of cardiovascular mortality/stroke (including subtypes) morbidity than conventional BP measurements.^19^^-^^21^ Systolic home BP was a betterpredictor of cardiovascular mortality than diastolic home BP.^22^

### Number of Home Blood Pressure Measurements Relative to the Predictive Power of Stroke

An increased number of measurements improved the predictive values of home BP. No threshold for the number of home BP measurements within the range of 1-14 measurements was evident for increasing the predictive power of stroke risk. Interestingly, even the initial home BP values (1 measurement) were significantly more closely related to stroke risk than conventional BP values (mean of 2 measurements).^20^ These results suggest that in addition to the number of measurements, other factors such as the absence of the white-coat effect are associated with the superior predictive power of home BP measurements.

### Use of Guidelines for Predicting Stroke Using Home Blood Pressure

Guidelines based on individualized categorizations, such as the 2003 European Society of Hypertension – European Society of Cardiology (ESH-ESC) guidelines,^23^ are more useful for predicting stroke^24^ than those based on simple BP-oriented categorizations, such as those of the Joint National Committee (JNC)-7.^25^^,^^26^ Home BP increased the predictive power of guideline categorizations compared with conventional BP.

### Prediction of Stroke by Home "Morning" versus "Evening" Blood Pressure Values

Home BP in the morning (morning BP) and in the evening (evening BP) provided equally useful information for stroke risk, whereas morning hypertension, which is observed specifically in the morning, might be a good predictor of stroke, particularly among individuals using anti-hypertensive medication.^27^

### White Coat Hypertension as a Transient State to Hypertension outside Medical Settings

Individuals with white-coat hypertension whose home BP was normal but whose conventional BP was high had an approximately 3-fold higher risk of eventually manifesting sustained home hypertension after 8 years compared with those having sustained normotension, indicating that patients with white-coat hypertension should be carefully monitored.^28^

### Predictive Values of Home Heart Rate

The advantages of home BP were also applicable to resting heart rate values assessed at home using a device designed for home BP measurement. Home heart rate was significantly associated with the risk of cardiovascular mortality. This relationship was statistically significant after adjustment for home BP values, indicating that home BP and heart rate represent simple sources of useful clinical information with which to assess cardiovascular risk.^29^

## CONCLUSION

In the Ohasama study, we demonstrated that ambulatory BP and home BP variabilities provide a variety of useful prognostic information. Ambulatory BP and home BP are useful tools with which to examine the prognostic significance of BP variabilities in clinical and epidemiologic settings. These variabilities also bias the diagnosis and treatment of hypertension in that the phasic, as well as tonic component of BP, must be considered for effective management of hypertension.
